# Imaging Procedure and Clinical Studies of [^18^F]FP-CIT PET

**DOI:** 10.1007/s13139-024-00840-x

**Published:** 2024-01-17

**Authors:** Changhwan Sung, Seung Jun Oh, Jae Seung Kim

**Affiliations:** Department of Nuclear Medicine, Asan Medical Center, University of Ulsan College of Medicine, 88, Olympic-Ro 43-Gil, Songpa-Gu, Seoul, 05505 Republic of Korea

**Keywords:** 2-carbomethoxy-8-(3-fluoropropyl)-3-(4-iodophenyl)tropane, [^18^F]FP-CIT, Dopamine transporter imaging, Positron-Emission Tomography, Parkinsonian Disorders

## Abstract

N-3-[^18^F]fluoropropyl-2β-carbomethoxy-3β-4-iodophenyl nortropane ([^18^F]FP-CIT) is a radiopharmaceutical for dopamine transporter (DAT) imaging using positron emission tomography (PET) to detect dopaminergic neuronal degeneration in patients with parkinsonian syndrome. [^18^F]FP-CIT was granted approval by the Ministry of Food and Drug Safety in 2008 as the inaugural radiopharmaceutical for PET imaging, and it has found extensive utilization across numerous institutions in Korea. This review article presents an imaging procedure for [^18^F]FP-CIT PET to aid nuclear medicine physicians in clinical practice and systematically reviews the clinical studies associated with [^18^F]FP-CIT PET.

## Introduction

The dopamine transporter (DAT) is situated at the terminals of dopaminergic neurons, where it facilitates the reuptake of released dopamine within the synapse. In Parkinson's disease (PD) patients, progressive dopaminergic neuronal degeneration projecting from the substantia nigra in the midbrain to the striatum is a crucial aspect of the pathophysiology leading to the development of movement symptoms. DAT imaging is widely recognized as a biomarker that effectively mirrors this process. Moreover, several radiopharmaceuticals have been developed for DAT imaging to detect dopaminergic neuronal degeneration in patients with parkinsonian syndrome, including [^123^I](1R)-2ß-Carbomethoxy-3ß-(4-iodophenyl)tropane ([^123^I]ß-CIT), [2-[[2-[[[3-(4-chlorophenyl)-8-methyl-8-azabicyclo[3,2,1]oct-2-yl]methyl](2-mercaptoethyl)amino]ethyl]amino]ethanethiolato(3-)-N2, N2′, S2, S2′]oxo-[1R-(exo–exo)-[ ^99m^Tc]technetium ([^99m^Tc]TRODAT-1), [^123^I]-2β-carbomethoxy-3β-(4-iodophenyl)-N-(3-fluoropropyl) nortropane ([^123^I]FP-CIT), N-3-[^18^F]fluoropropyl-2β-carbomethoxy-3β-4-iodophenyl nortropane ([^18^F]FP-CIT), [^11^C]N-(3-iodoprop-2E-enyl)-2β-carbomethoxy-3β-(4-methyl-phenyl)nortropane ([^11^C]PE2I), and [^18^F]-(E)-N-(3-iodoprop-2-enyl)-2β-carbofluoroethoxy-3β- (4′-methyl-phenyl) nortropane ([^18^F]FE-PE2I).

Among these, [^123^I]β-CIT exhibits high affinity for the DAT and low non-specific binding. However, it is characterized by slow pharmacokinetics in the brain, with imaging typically performed at 18–24 h post-injection. Its usage has been restricted since 2000 due to strengthened regulations by the Food and Drug Administration [1]. [^99m^Tc]TRODAT-1 has been used in Taiwan since 2005 after it was granted approval, but it shows relatively slow pharmacokinetics and has a low specific/non-specific uptake ratio [2]. [^123^I]FP-CIT, a widely used DAT imaging agent in Europe and the USA, was approved for DAT single photon emission computed tomography (SPECT) imaging in Europe, the USA, and Korea. [^18^F]FP-CIT was approved by the Ministry of Food and Drug Safety (MFDS) of Korea, in 2008, as the first radiopharmaceutical for positron emission tomography (PET) imaging and has been widely utilized in Korea. [^18^F]FP-CIT is a compound in which a fluoroalkyl group is incorporated into the tropane ring of ß-CIT. When the affinity to DAT was measured in vitro, the Ki value of [^18^F]FP-CIT was 3.50 ± 0.39 nM, which is similar to that of ß-CIT (1.40 ± 1.40 nM), and exhibited relatively fast pharmacokinetics in vivo [3]. [^18^F]FP-CIT has no lipophilic labeled metabolites and has a longer half-life than [^11^C], which is advantageous for imaging [4]. In a comparative study using [^123^I]ß-CIT in rats and pigs, [^18^F]FP-CIT PET exhibited a higher striatum-to-cortex binding ratio [5]. A head-to-head comparison study demonstrated no significant difference in the diagnostic accuracy of [^18^F]FP-CIT PET compared to [^123^I]FP-CIT SPECT. Furthermore, both radiotracers have consistently revealed decreased DAT binding in the caudate nucleus and putamen of patients with early PD compared to control subjects [6]. However, [^18^F]FP-CIT PET exhibited superior contrast in semi-quantitative analysis [7]. Additionally, [^18^F]FP-CIT PET offers the advantage of clinical convenience compared to [^123^I]FP-CIT SPECT, which requires thyroid blocking and longer imaging acquisition time.

This review paper introduces an imaging procedure for [^18^F]FP-CIT PET to assist nuclear medicine physicians in their clinical work. Additionally, it systematically examines the clinical studies about [^18^F]FP-CIT PET.

## Part 1: Imaging Procedure

### Patient Preparation and Precautions

Fasting or control of blood glucose levels was not required. A history of drug administration that may affect striatal DAT binding was obtained, and the drugs were discontinued if clinically feasible. Central nervous system stimulants such as amphetamine, cocaine, and methylphenidate exhibit a strong affinity for the DAT, reducing DAT binding. It is advisable to discontinue these stimulants for a period equivalent to at least five half-lives to ensure a significant decrease in their effect. Modafinil, cannabidiol, ephedrine, phentermine, antidepressants (bupropion, radafaxine), anesthetics (ketamine, phencyclidine, and isoflurane), opioids (fentanyl), and anticholinergics (benzatropine) can also reduce striatal DAT binding of [^18^F]FP-CIT, although to a lesser degree. Sertraline and chronic lithium treatment may also affect it. Moreover, drugs affecting gastrointestinal motility such as metoclopramide, levosulpiride, clebopride can also affect striatal DAT binding of [^18^F]FP-CIT and are common causes of drug-induced parkinsonism (DIP) [8–10]. Anti-parkinsonian drugs (L-dopa, dopamine agonists, N-methyl-D-aspartate receptor blockers, monoamine oxidase B inhibitors, and catechol-o-methyltransferase inhibitors) do not substantially affect DAT binding at standard doses, so it is not necessary to discontinue their usage [11, 12].

[^18^F]FP-CIT PET is contraindicated in pregnant or breastfeeding women. It is necessary to consider the possibility of pregnancy and lactation in fertile women, and if confirmed, the nuclear medicine physician's decision on whether to implement it is required.

### Radiopharmaceutical

There were several reports on the synthesis of [^18^F]FP-CIT. The previous synthesis method has two-step procedures: [^18^F]fluorination for ditosyl propane and N-alkylation between nor-β-CIT and [^18^F]fluoropropyl tosylate. However, this procedure needs a long reaction time for alkylation and shows a very low yield, such as 2–4% [4]. Chaly et al. introduced a one-step preparation method for [^18^F]FP-CIT from a precursor. Unfortunately, this approach also demonstrated a very low yield, approximately 1–2%, posing a significant challenge and impeding the routine clinical application of [^18^F]FP-CIT [13]. Lee et al. developed a fully automatic synthesis method with various kinds of protic solvents, such as [^18^F]fluorination solvents. They showed higher yields of 10–20%. Consequently, these results initiated clinical trials of [^18^F]FP-CIT in Korea [14]. After phase III clinical trials, Asan Medical Center received new drug approval for [^18^F]FP-CIT from the MFDS of Korea in 2008. Lee et al. persevered in optimizing the [^18^F]fluorination conditions for [^18^F]FP-CIT, addressing challenges encountered in prior productions that disrupted stable and consistent manufacturing processes. After controlling the acidic conditions during [^18^F]fluorination, they achieved a highly stable radiochemical yield suitable for routine clinical applications [15, 16].

A dose of 5 mCi (185 MBq) of [^18^F]FP-CIT diluted with normal saline is recommended for intravenous injection for PET imaging. Clinical indications and dosage for pediatric patients are currently not established.

### Imaging Acquisition

#### Timing

**Fig. 1:**
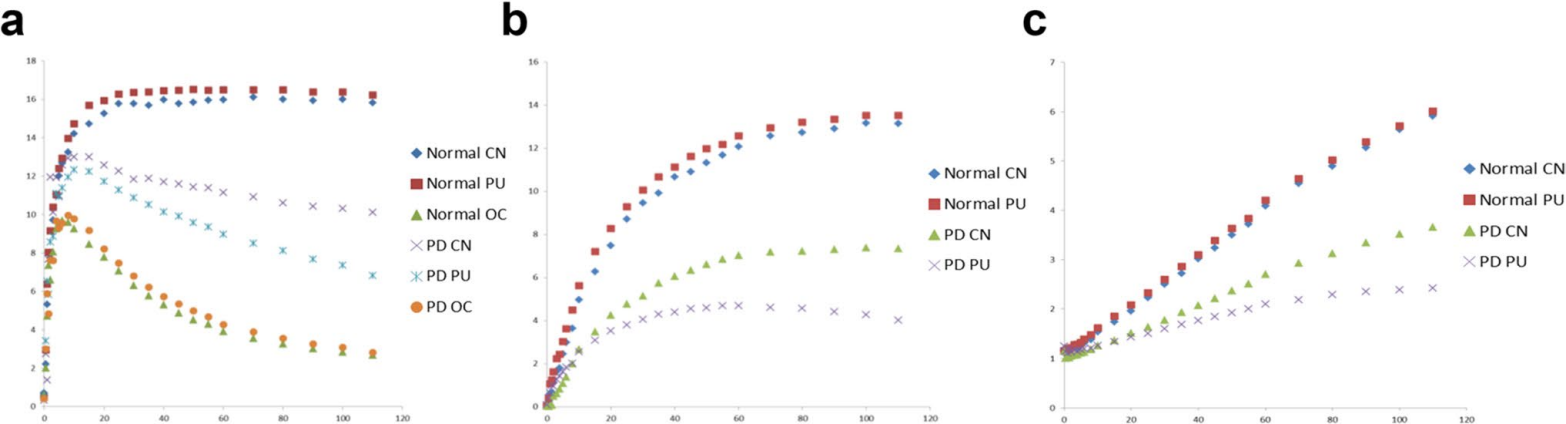
Time activity curve (TAC) of [^18^F]FP-CIT. Two hours of dynamic [^18^F]FP-CIT PET performed in six patients with Parkinson's disease and four healthy controls showed different mean uptake values (**a**) specific uptake values (**b**), and specific to non-specific uptake ratio (**c**) of caudate nuclei (CN), putamen (PU), and occipital cortex (OC, as a reference)

The specific uptake of [^18^F]FP-CIT reaches its peak at 90–120 min in the striatum of normal individuals. Furthermore, this accurately distinguishes normal individuals from those with PD as much as the binding potential measured by pharmacodynamic analysis [17, 18]. However, [^123^I]FP-CIT SPECT images are usually obtained at least 3 h after the injection. Given their analogous molecular structures, this timing aligns with that of [^18^F]FP-CIT. The rationale is that the specific/non-specific binding ratio in the striatum gradually escalates to 3 h, eventually reaching a relatively stable state [19]. [^18^F]FP-CIT PET is also expected to reach a pseudo-equilibrium state after 2 h, similar to [^123^I]FP-CIT, so it would be better to acquire images at the same uptake time as [^123^I]FP-CIT (Fig. 1). Currently, several centers for nuclear medicine imaging are acquiring images at 90 to 120 min after injection for patient convenience. A report showed that the specific/non-specific uptake ratio of the striatum analyzed in the 90-min delay image is as good in differentiating PD from normal subjects as the binding capacity obtained by dynamic analysis [20]. However, achieving a pseudo-equilibrium state within 90 min might not be consistent across all patients. Moreover, the 90-min image is more susceptible to hemodynamic factors influenced by the patient's condition than the 3-h image. Thus, if feasible, obtaining delayed images at 2 or 3 h post-injection is advisable to ensure a more reliable representation of the specific/non-specific binding ratio.

Regarding early phase imaging, the uptake of FP-CIT in the brain cortex and striatum is known to rapidly increase during the initial 15 min due to a high first-pass influx rate (*K*_1_) [17]. Early imaging immediately after the injection of radiotracer with scanning time of 5 min, 7 min, and 10 min with [^18^F]FP-CIT PET demonstrated high correlations (range of R value, 0.54 – 0.87) in quantitative analysis across the putamen, cerebellum, midbrain, and superior frontal cortex when compared with [^18^F]FDG PET. No significant differences were observed between the scanning time [21]. Signal-to-noise ratio were acceptable level for all the early imaging. The contrast in the putamen gradually increased, while the contrast in the cerebellum exhibited a decreasing pattern. Considering that specific dopaminergic uptake becomes dominant after 10 min [17, 21], it is advisable to perform early imaging within the first 10 min, tailored to the PET system environment of each center.

#### Position and Imaging

The back of the patient's head is placed on the table in the supine position with the orbitomeatal line and the mid-sagittal plane at right angles to the table. The patient's head should be oriented towards the gantry using a headrest. First, a CT scan for attenuation correction is conducted, followed by PET imaging. Given its higher sensitivity, a window of 5–7 min for image acquisition is deemed appropriate for digital PET/CT. However, in the case of a conventional PET/CT system, a longer image acquisition time of 10 min is recommended to ensure optimal image quality [22].

#### Early Perfusion Imaging

For the early perfusion phase imaging, an intravenous line is required before the scanning procedure. After the acquisition of CT images for attenuation correction, the PET image is acquired in the list mode by slow bolus injection of a radiotracer. Approximately 10 mL of normal saline is flushed after the radiotracer injection. The perfusion in the early phase imaging refers to the cerebral blood flow rather than tissue-extracted perfusion. In other words, while the extraction fraction of FP-CIT is not particularly low at 0.65 [17], early perfusion imaging is significantly influenced by vascular flow rather than tissue-extracted perfusion. Even though there may be regional differences between flow and metabolism, it is generally known to correlate with regional metabolism in the brain [23]. So, additional early perfusion imaging is perfusion-proxy imaging, which was helpful for the differential diagnosis of parkinsonism [21, 24, 25]. Early [^18^F]FP-CIT PET imaging is known to exhibit similar findings to early imaging of other radiotracers or tracers that reflect perfusion or metabolism, such as [^99m^Tc]ECD SPECT [26], [^18^F]FDG PET [21, 27, 28]. Early imaging of [^18^F]flutemetamol PET, one of the amyloid imaging agents, also demonstrated a similar pattern to that of [^18^F]FP-CIT PET [29]. Furthermore, investigations into early perfusion imaging have unveiled associations with non-motor symptoms in PD. For instance, in patients with PD with impulse control disorders (ICD), early perfusion imaging revealed increased uptake in the left insular and right posterior cingulate cortex and decreased uptake in the bilateral ventral pallidum, compared to patients with PD without ICD. Additionally, there was a notable decrease in ventral striatum uptake in delayed imaging for patients who have ICD [30]. Similarly, increased perfusion in the left posterior cingulate on early imaging in patients with sleep disturbances, a common non-motor symptom in PD, is often associated with apathy [31].

One of the most valuable clinical applications of early perfusion imaging lies in its capacity to provide additional insights into the differential diagnosis of parkinsonism. Please refer to the "Diagnosis of PD and Differential Diagnosis of Parkinsonism" section for more detailed information.

### Reconstruction and Processing

Acquired PET data undergoes reconstruction, incorporating corrections for scatter, attenuation, random coincidences, and sampling non-uniformity. Two-dimensional and three-dimensional (3D) data acquisition modes typically utilize iterative reconstruction algorithms rather than conventional filtered back projection. When a reconstruction system supports the time-of-flight algorithm, it has the potential to substantially enhance image quality. In the context of quantitative analysis, it is strongly recommended to standardize parameters such as subsets and iterations, consistent with those previously applied to a normal database. Optimal parameters may vary depending on the specific PET/CT system, so it is recommended to consult the manufacturer's recommendations. Generating both attenuation-corrected PET images using the acquired CT data and non-attenuation-corrected PET images is recommended in the reconstruction process.

### Visual Interpretation

Attenuation-corrected PET images are typically displayed in the coronal, sagittal, and transaxial planes. Furthermore, including rotating maximum-intensity-projection (MIP) images as part of imaging protocol is beneficial.

**Fig. 2:**
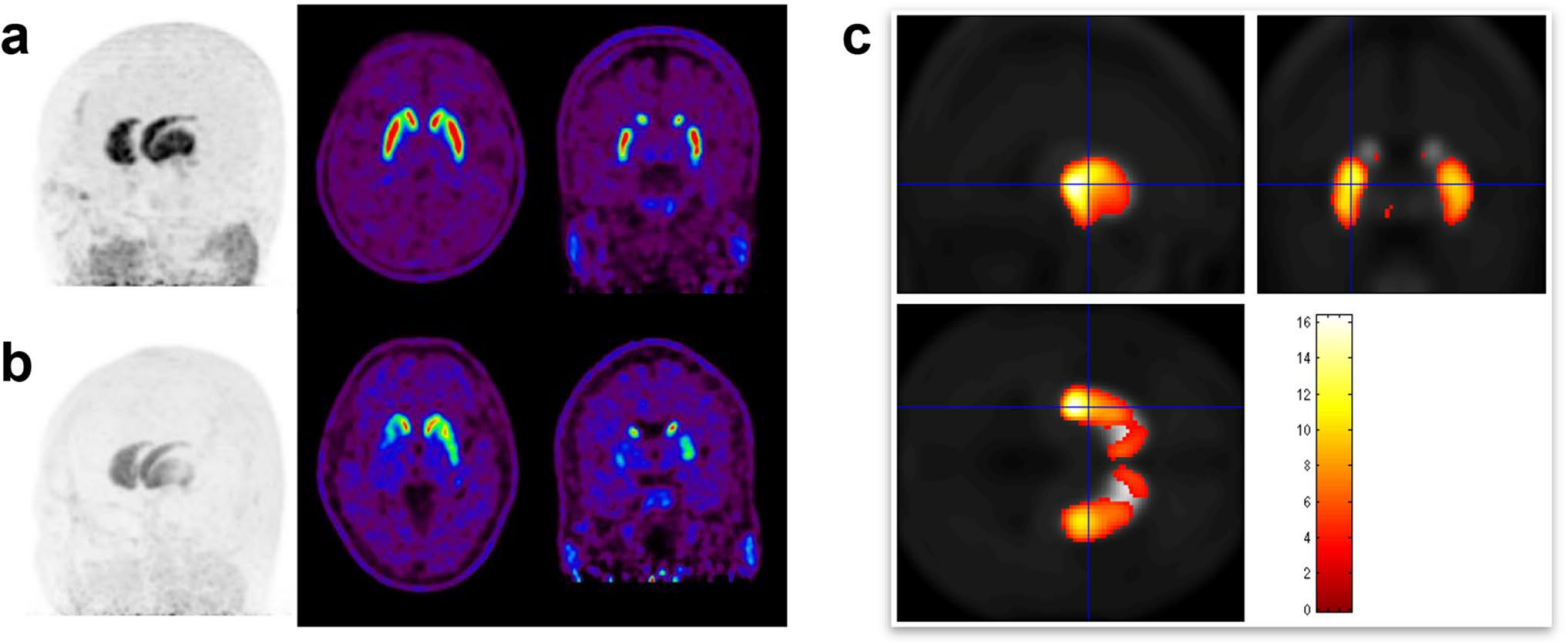
Typical regional striatal [^18^F]FP-CIT uptake pattern of Parkinson's disease (PD). Maximum-intensity-projection, transaxial, and coronal images show symmetric and homogenous striatal uptake in normal subjects (**a**) but predominantly reduced striatal uptake in the posterodorsal portion of the putamen in patients with PD (**b**). In the voxel-wise SPM analysis (**c**), the PD group shows an asymmetric decrease in striatal uptake compared to the normal group, with a severely decreased uptake in the posterodorsal putamen (uncorrected p-value < 0.001)

**Fig. 3:**
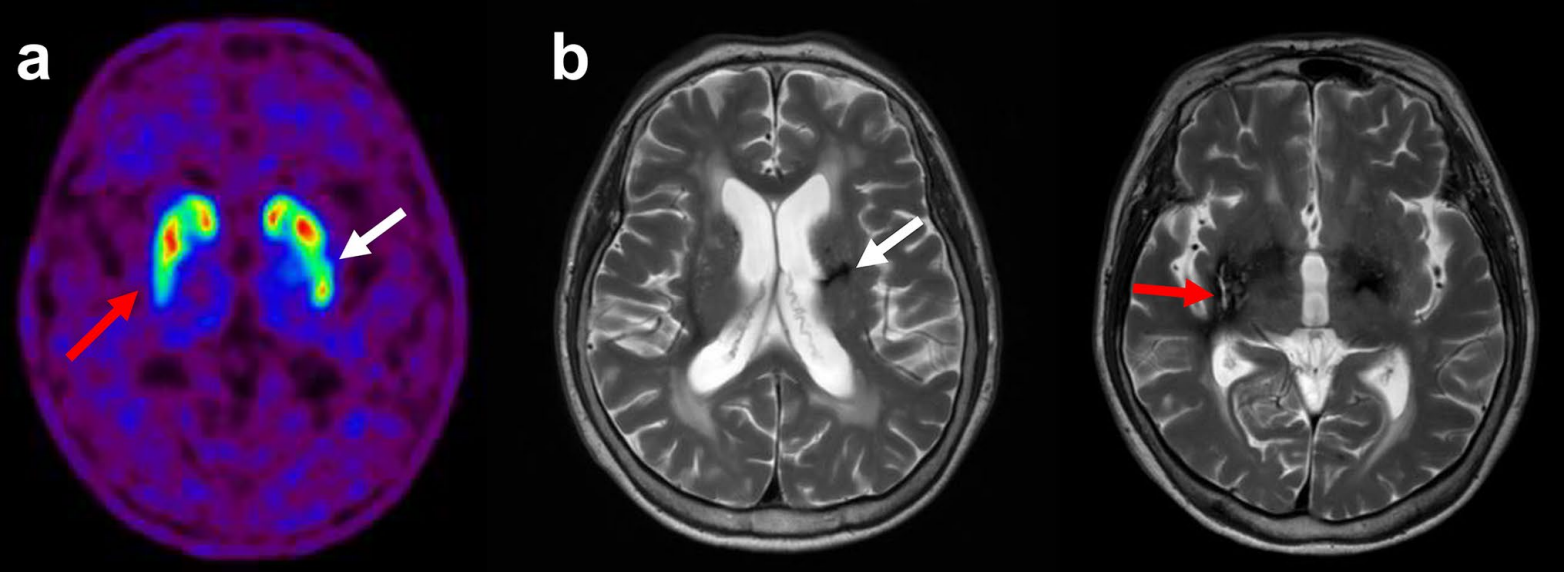
Representative images of vascular parkinsonism with ischemic lesions-related changes. Transaxial [^18^F]FP-CIT PET (**a**) shows focally reduced uptake in bilateral putamens with corresponding an old striatocapsular infarction (white arrows) and an old hemorrhagic infarction (red arrows) on T2 weighted MR images (**b**)

To visually analyze [^18^F]FP-CIT PET images in color scale, it is essential first to set up appropriate window settings [32]. Integrated reading of brain PET images, including transaxial images aligned parallel to the anterior commissure – posterior commissure line and coronal, sagittal, and MIP images, is recommended. MIP images provide the overall contour and the activity of the striatum. Typical image findings of normal subjects exhibit rabbit-like uptake on MIP images with symmetric and homogenous DAT binding of both strata (Fig. 2a). If abnormalities are detected, it is essential to determine the location of the lesions, assess asymmetry, and discern whether reduced DAT binding with [^18^F]FP-CIT is diffuse or focal. It is worth noting that the uptake of the caudate nucleus may be reduced in cases of ventricle dilatation due to normal pressure hydrocephalus (NPH) or cerebral cortical atrophy. In general, if abnormalities in the striatum are found, it should be determined whether it is due to local anatomical lesions such as ischemic lesions (Fig. 3) or degenerative parkinsonism such as PD or atypical parkinsonism (APD).

**Fig. 4:**
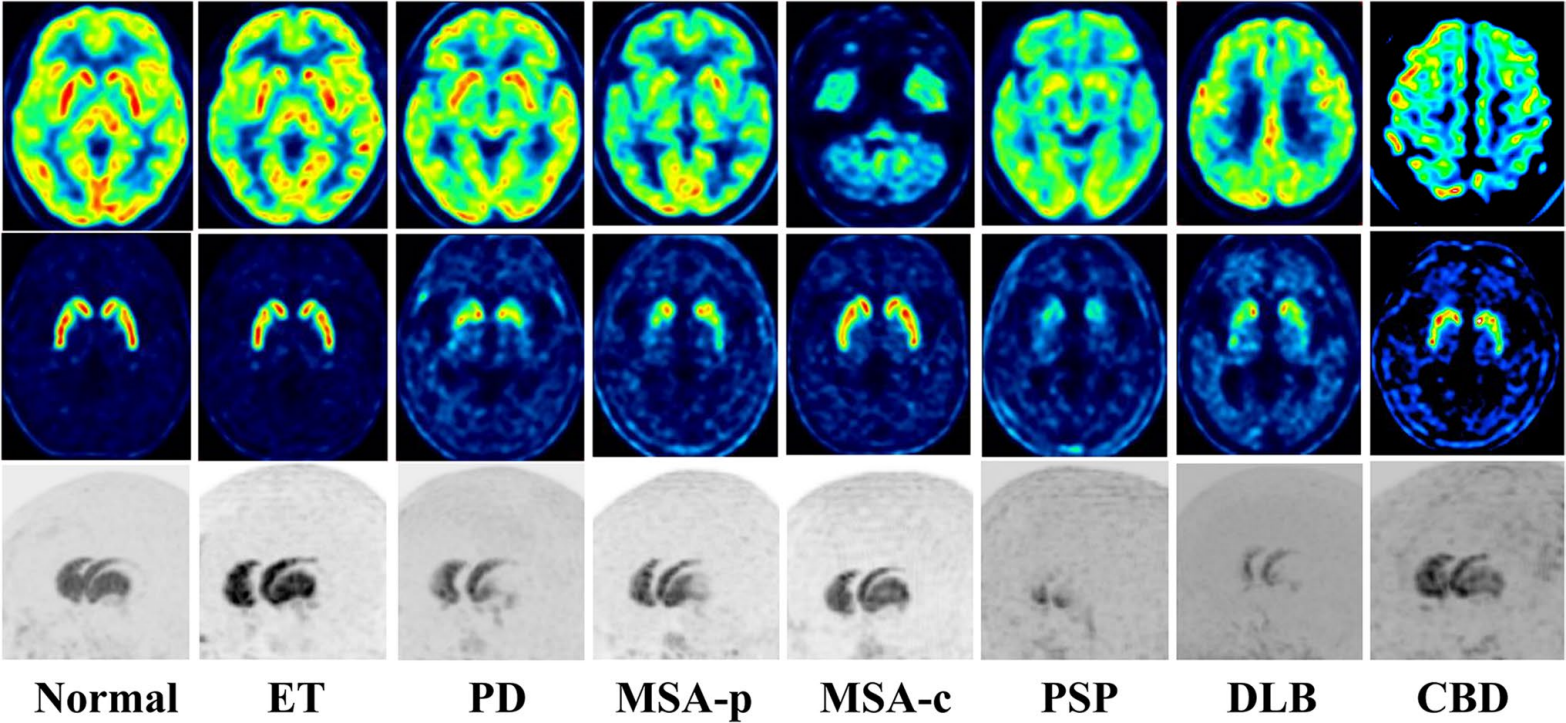
Representative dual-phase [^18^F]FP-CIT PET images in normal subjects and patients with parkinsonism. Transaxial early (top), delay (middle), and maximum-intensity-projection (bottom) images show different image findings according to the disease. ET; essential tremor, PD; Parkinson’s disease, MSA-P; multiple system atrophy parkinsonian subtype, MSA-C; multiple system atrophy cerebellar subtype, PSP; progressive supranuclear palsy, DLB; dementia with Lewy body, CBD; corticobasal degeneration

Ischemic lesions frequently detected in older adults can affect various striatum regions and tend to be randomly distributed rather than symmetrically located. Ischemic lesions of the nigrostriatal dopaminergic tract, rather than the striatum, may cause a reduction in striatal DAT binding more broadly than in the actual lesion. Diffusely reduced striatal uptake may be shown in patients with ischemic lesions in the subcortical white matter. Correlative imaging interpretation with CT or magnetic resonance imaging (MRI) is essential, especially for diagnosing vascular parkinsonism (VaP). Degenerative parkinsonism demonstrates a subregional preference pattern based on the underlying neurodegenerative disease. PD typically exhibits a predominantly reduced uptake in the posterior putamen, especially the dorsal portion, although early stages may present with asymmetrically reduced uptake of the posterior putamen (Fig. 2b). In early stages of PD, the contour and uptake of the caudate nucleus tend to remain relatively preserved, and ventral striatum uptake is usually retained. Multiple system atrophy, especially the parkinsonian subtype (MSA-P), may exhibit a predominantly decreased uptake in the posterior putamen, similar to PD. Multiple system atrophy cerebellar subtype (MSA-C) may appear normal on the delayed imaging but could exhibit asymmetrically decreased cerebellar uptake of the cerebellum if early perfusion imaging is available. Compared to other degenerative parkinsonism, progressive supranuclear palsy (PSP) or pure akinesia with gait freezing (PAGF) may show severely decreased uptake of the entire striatum, especially with the early involvement of the caudate nucleus. Corticobasal degeneration should be considered in patients with clinical symptoms of dystonic movements when the reduced striatal binding is asymmetric. Representative images and imaging features in [^18^F]FP-CIT PET for the different types of parkinsonism are presented and described in Fig. 4 and Table 1, respectively. However, the imaging features presented in the table exhibit overlaps between diseases, and caution is warranted when applying them to individual patients. Particularly in PD patients, there have been numerous reports on clinical and imaging heterogeneity [33–35], and special considerations are required regarding the asymmetry. In the "Body-first" type based on the Gut-brain axis theory, aggregation of a-synuclein and Lewy body deposition first occurs in the vagus and sympathetic nerves of the gut, with subsequent propagation to the central nervous system exhibiting a more symmetric pattern. In contrast, the "Brain-first type" shows prominent asymmetry in clinical symptoms and imaging findings due to the highly lateralized connectome of the CNS system [36]. In other words, the degree of asymmetry in PD patients may vary depending on whether they belong to the Body-first type or Brain-first type.

**Table 1:** Imaging features in early and delayed phase [^18^F]FP-CIT PET for the different causes of parkinsonism

Type of parkinsonism	Early				Delayed							
	Cerebral cortex	Cerebellum	Mid-brain	Striatum	VS	CN	AP	PP	Asymmetry	APG	VDG	CP ratio
Non-DP (ET or DIP)	−	−	−	−	−	−	−	−	−	−	−	−
VaP	−	−	−	− /↓	−	−	−	−	−	−	−	−
IPD	− /↓	−	−	− /↑	− /↓	− /↓	↓↓	↓↓↓	+ +	↑↑↑	↑↑↑	↑↑↑
MSA-P	−	↓/ −	−	↓↓	− /↓	− /↓	↓	↓↓	+ +	↑↑	− /↑	↑
MSA-C	−	↓↓	−	↓	− /↓	− /↓	− /↓	− /↓	+	− /↑	− /↑	−
PSP	Medial frontal	−	↓/ −	↓	− /↓	↓↓↓	↓↓↓	↓↓↓	−	− /↑	− /↑	−
CBD	PMC, SMC	−	−	↓	− /↓	− /↓	↓	↓↓	+ + +	− /↑	− /↑	↑

Non-DP, Non-degenerative parkinsonism; ET, essential tremor; DIP, drug-induced parkinsonism; VaP, vascular parkinsonism; IPD, idiopathic Parkinson's disease; MSA-P, multiple system atrophy parkinsonian type; MSA-C, multiple system atrophy cerebellar type; PSP, progressive supranuclear palsy; CBD, corticobasal degeneration; VS, ventral striatum; CN, caudate nucleus; AP, anterior putamen; PP, posterior putamen; APG, anteroposterior gradient; VDG, ventrodorsal gradient; CP ratio, caudate nucleus putamen ratio; PMC, primary motor cortex; SMC, supplementary motor cortex

**Fig. 5:**
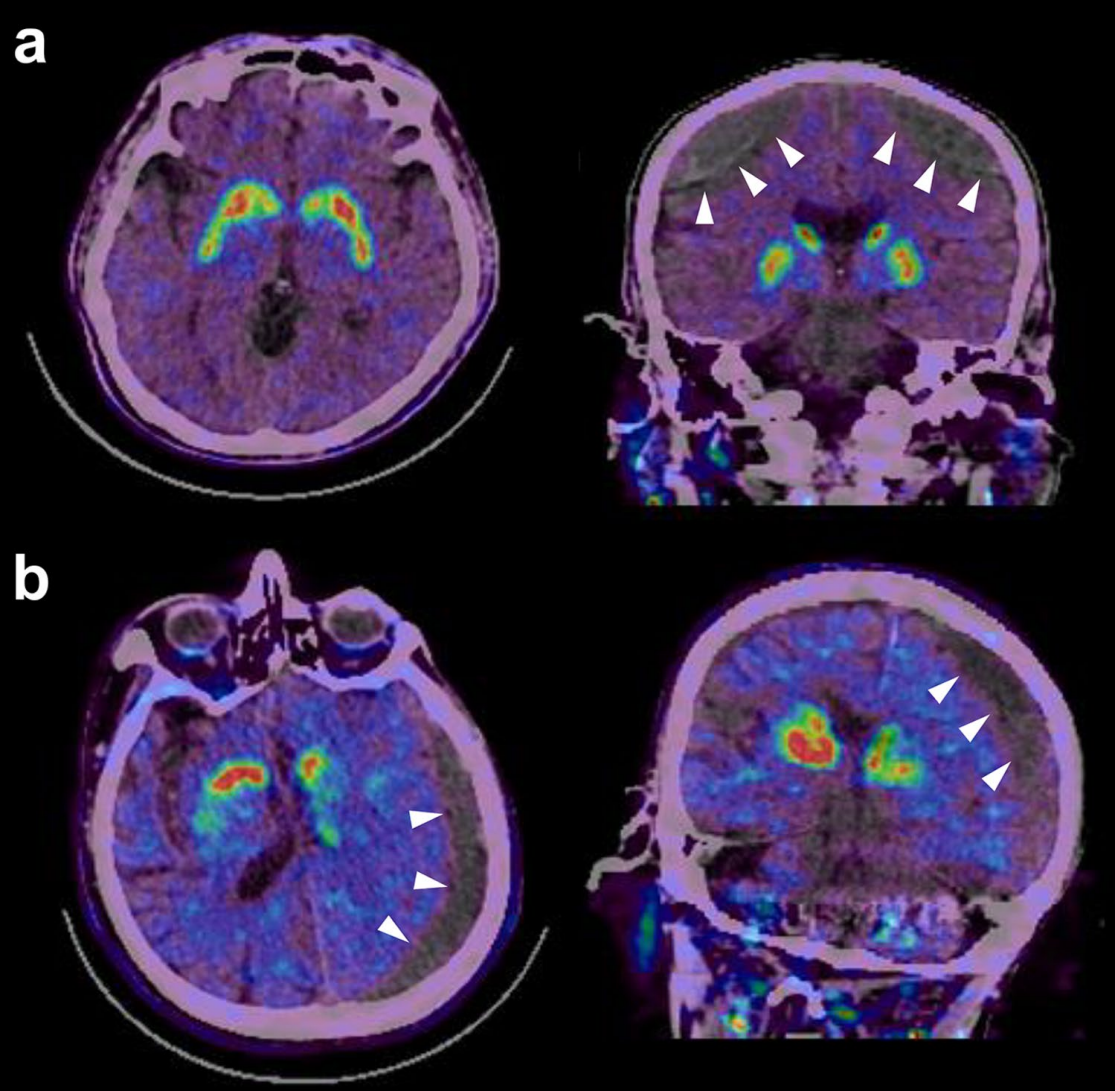
(**a**) Representative cases with subdural hematoma detected by [^18^F]FP-CIT PET/CT performed for initial evaluation of parkinsonism. (**a**) Transaxial and coronal [^18^F]FP-CIT PET/CT fusion images of a 73-years old man who complained of gait disturbance and bradykinesia after a traffic accident one month ago show normal striatal uptake and subdural hematomas (arrowheads) in the bilateral frontal convexity. (**b**) Transaxial and coronal [^18^F]FP-CIT PET/CT fusion images of a 74-years old man who complained of frequent falls and gait disturbance show reduced striatal uptake and also subdural hematoma (arrowheads) in the left parieto-temporal convexity

Among patients presenting with parkinsonism, subdural hematoma caused by previous trauma can mimic degenerative parkinsonism (Fig. 5a), or undetected subdural hematoma in patients who frequently fall due to neurodegenerative disease is occasionally found in [^18^F] FP-CIT PET/CT (Fig. 5b), so it is crucial to examine combined CT carefully.

Finally, when abnormalities in striatal uptake are suspected but are not typical findings of degenerative parkinsonism (e.g., combined ischemic lesions and early stage of PD) or the patients with clinically typical PD appear to have normal findings, follow-up [^18^F]FP-CIT imaging after 1–2 years is advisable, and quantitative analysis can provide additional information because degenerative parkinsonism shows rapid progression of DAT loss compared to non-degenerative parkinsonism [37, 38].

### Quantification

Quantitative analysis can give additional information when visual interpretation is equivocal in the clinical setting. Various methods for quantitative analysis of [^18^F]FP-CIT PET have been studied and proposed [39–43]. While comparing different kinetic and semi-quantitative methods, the simplified reference tissue model showed the highest precision, accuracy, and good contrast between patients and control groups compared to the complete reference tissue model, SUV, and SUVr [18]. However, the specific to non-specific uptake ratio is the most commonly used semi-quantitative outcome measure in clinical and research settings because most institutions do not employ dynamic [^18^F]FP-CIT PET imaging method due to the relatively long pharmacokinetics of [^18^F]FP-CIT and patient's convenience. The specific to non-specific uptake ratio for [^18^F]FP-CIT is calculated by determining the ratio of radiotracer uptake in the striatal target region to that in a background region, typically the occipital cortex. This ratio is computed by carefully positioning the volume of interest (VOI).

For the appropriate and reproducible positioning of VOIs, [^18^F]FP-CIT PET template-based method was reported to show high agreement with MR-based and manual methods for spatial normalization [40] and spatial normalization and quantitative analysis using combined CT of the PET/CT system were also reported to be comparable with MR-based method. [41]. Spatial normalization using early perfusion imaging has been studied recently, and quantitative analysis reported higher intraclass correlation coefficients (early image-based, 0.980; delayed image-based, 0.895) with MRI-based methods than the results obtained from delayed imaging. [43].

Volumetric analysis using VOI techniques accurately distinguished between PD and healthy subjects, correlating well with various clinical parameters [42]. Quantitative analysis of [^18^F]FP-CIT PET images generally employs several subregional VOIs of the striatum, such as the caudate nucleus, anterior and posterior putamen, and ventral striatum. In high-resolution PET images, small target regions in the brain stem, such as substantia nigra and dorsal raphe nuclei, can be used.

Quantitative analysis can provide a more precise assessment of striatal DAT, allowing for more reproducible measurements of disease and response to therapy. Evaluation of test–retest variability is necessary for accurate quantification and comparison of serial studies, and [^18^F]FP-CIT PET showed low variability and high reliability. DAT binding of the posterior putamen showed lower variability in the automated method than in the manual method [44]. Further studies are needed to establish standardized quantitative analysis methods and a normal range for each age group suitable for variable clinical environments, including PET systems and software.

### Dosimetry

In adults, the absorbed dose of [^18^F]FP-CIT in the brain was 8.11 × 10^–3^ mGy/MBq and the effective dose was 1.20 × 10^–2^ mSv/MBq, which was lower than 0.23 mGy/MBq for the striatum and 0.024 mSv/MBq of [^123^I]FP-CIT, commonly used radiopharmaceutical for DAT SPECT imaging [45, 46]. The urinary bladder (0.0586 ± 0.0164 mGy/MBq) [45] or the basal ganglia (0.029—0.069 mGy/MBq) [47] appeared as critical organs but within the acceptable range in humans.

## Part 2: Clinical Studies

### Diagnosis of PD and Differential Diagnosis of Parkinsonism

[^18^F]FP-CIT PET has been widely used in Korea to diagnose parkinsonism. The label indication for [^18^F]FP-CIT involves assessing striatal DAT density in patients with confirmed or suspected clinical symptoms and signs of parkinsonism through other clinical tests. The phase III clinical trial to evaluate the efficacy and the safety of [^18^F]FP-CIT PET in PD and essential tremor (ET) patients showed high diagnostic sensitivity and specificity (100% and 97.4% for visual analysis, 97.4% and 100% for quantification, respectively). In this study, the uptake of contralateral putamen in patients with PD showed the highest diagnostic accuracy (area under the curve [AUC], 0.997; 95% confidence interval [CI], 0.989–1.004; p < 0.001), followed by ipsilateral putamen (AUC, 0.975; 95% CI, 0.948–1.002; p < 0.001), contralateral caudate (AUC, 0.904; 95% CI, 0.837–0.971; p < 0.001), and ipsilateral caudate (AUC, 0.849; 95% CI, 0.764–0.934; p < 0.001) [48].

In the quantitative analysis study of [^18^F]FP-CIT PET, it was observed that DAT binding in the PD group was reduced by 70% in the contralateral posterior putamen compared to normal levels, aligning with the clinical symptoms. Moreover, the ipsilateral putamen exhibited a significant albeit lesser decrease, particularly in the posterior regions [49]. [^18^F]FP-CIT PET is beneficial for differential diagnosis of parkinsonism by non-presynaptic degeneration, such as ET and DIP [50]. It can be used to determine whether nigrostriatal dopaminergic neuronal degeneration is accompanied when postural tremor and resting tremor are combined [51]. VaP is a relatively common cause of parkinsonism, especially in Korea, and [^18^F]FP-CIT PET helps differentiate VaP from PD [52]. Clinical manifestations of VaP vary depending on where the ischemic lesions occur in the brain [53], and it may appear like atypical parkinsonism such as PSP [54]. Even in the case of VaP with visually normal-looking DAT binding on [^18^F]FP-CIT PET who did not have ischemic lesions on the striatum and only had subcortical white matter hyperintensities revealed reduced DAT binding in most striatal subregions compared to normal controls [55]. The number and volume of white matter hyperintensities evaluated by fluid-attenuated inversion recovery MRI were correlated with DAT binding of the caudate nucleus in [^18^F]FP-CIT PET [56]. [^18^F]FP-CIT PET is also helpful for differential diagnosis of atypical parkinsonism with improved resolution compared to SPECT. In the quantitative analysis, the sensitivity and specificity to distinguish between PSP and PD using the inter-subregional ratio (ISR) of the anterior caudate/ventral striatum were 94% and 92%, respectively. The sensitivity and specificity to distinguish between MSA and PD using ISR of posterior putamen/ventral putamen were 90% and 45%, respectively [57]. There were differences in striatal DAT binding patterns on [^18^F]FP-CIT PET image between subtypes of MSA, and striatal DAT binding in cases of the MSA-P type was more severely reduced with reduced ISRs of posterior putamen/anterior putamen and anterior putamen/ventral striatum than the MSA-C type [58]. PAGF showed a subregional striatal DAT binding pattern similar to PSP with a substantially reduced caudate nucleus on [^18^F]FP-CIT PET, suggesting a high possibility of being a phenotypic variant of PSP [59, 60]. In the comparison study of frontotemporal dementia, which may present clinically like PSP, the PSP group showed more reduced DAT binding than the frontotemporal dementia group in all striatal subregions with a prominent involvement in the caudate nucleus [61].

The combined use of [^18^F]FP-CIT PET and [^18^F]FDG PET is helpful for the differential diagnosis of atypical parkinsonism [62]. Dual-phase [^18^F]FP-CIT PET imaging is also useful for differentiating APD from PD since early perfusion phase images of [^18^F]FP-CIT PET show [^18^F]FDG PET-like imaging findings [27, 63]. Differential diagnosis using dual-phase [^18^F]FP-CIT PET was beneficial for MSA (81% MSA-P, 77% MSA-C) [25]. Corticobasal degeneration showed an asymmetric decrease in the contralateral side, more prominent in the delay phase, making it difficult to differentiate from PD. However, there has been a case report suggesting that dual-phase imaging was helpful in the differential diagnosis with reduced uptake in the contralateral side of the cerebral cortex on early imaging [64]. In the fulminant type of corticobasal syndrome, there is a case report showing decreased metabolism of the anterior frontal cortex, caudate nucleus, and contralateral cerebellum on [^18^F]FDG PET with complete unilateral loss of DAT binding on [^18^F]FP-CIT PET [65]. It is known that AD-related cognitive impairment patients with mild parkinsonism show different subregional patterns from dementia with Lewy bodies. AD-related cognitive impairment patients with mild parkinsonism show reduced DAT binding in the caudate nucleus but not in the posterior putamen, the most common and early involved subregion in dementia with Lewy body and patients with PD [66].

[^18^F]FP-CIT PET can also visualize serotonin transporter (SERT) uptake in the midbrain with a higher resolution than SPECT and can provide additional information for identifying the causes of parkinsonism. There was a notable decrease in midbrain and striatal uptake in idiopathic PD and MSA-P. However, MSA-P exhibited a more pronounced reduction in substantia nigra uptake than PD. MSA-C and DIP showed normal striatal uptake but a decreased uptake in the substantia nigra and dorsal raphe nucleus, and in VaP, there was a noticeable trend of reduced uptake in the substantia nigra without a concurrent decrease in the dorsal raphe nucleus [67]. Another common cause of parkinsonism is NPH. NPH patients mainly present with lower body parkinsonism but may also show marked asymmetric and upper body parkinsonism. [^18^F]FP-CIT PET in NPH patients can have normal findings [68] or mild and heterogeneously decreased uptake in the caudate nucleus rather than the putamen, unlike degenerative parkinsonism [69]. There was a case report of Wilson's disease presenting with head/hand tremors and dystonia, and T2 MRI showed signal changes in the basal ganglia but revealed normal findings on [^18^F]FP-CIT PET [70].

In summary, [^18^F]FP-CIT PET is useful in differentiating degenerative parkinsonism, such as PD, MSA, and PSP, from non-presynaptic parkinsonism, such as DIP, VaP, and ET. Furthermore, the subregional DAT binding pattern on high-resolution images of [^18^F]FP-CIT PET and early perfusion phase images are also helpful in differentiating PD from APD, such as MSA and PSP.

### Aging Effect and Longitudinal Follow-up of Parkinsonism with [^18^F]FP-CIT PET

[^18^F]FP-CIT PET has rarely been performed in pediatrics, but the longitudinal monitoring conducted in rats suggested the maturation of the dopaminergic system. The specific binding ratio of [^18^F]FP-CIT in the caudate, putamen, and nucleus accumbens gradually increased until 15 weeks of age, and there was no significant difference compared with rats at 20 weeks of age [71]. It is known that striatal DAT binding gradually decreases with aging in adults, and the aging effect appears differently in patients with PD and healthy controls on [^18^F]FP-CIT PET. In regression analysis according to age between patients with PD and healthy controls, there was no difference in the coefficient of the caudate nucleus between groups, but it exhibited a smaller age effect on the putamen in the PD group (-0.061 vs. -0.017) [72]. Striatal DAT binding assessed by [^18^F]FP-CIT PET seems to decrease in a non-linear pattern revealed by longitudinal follow-up of patients with PD and non-PD (ET or DIP). Both absolute annual reduction and relative annual reduction were more significant in PD. The absolute annual reduction of the posterior putamen showed a negative correlation with disease duration, but no significant correlation was found with relative annual reduction [37]. In another longitudinal follow-up study with 20 newly diagnosed patients with PD, DAT binding in the putamen decreased by 4.2% per year; however, there was no significant change in the caudate nucleus during 30 months of follow-up [73]. The relationship between the age of onset and [^18^F]FP-CIT PET pattern has also been reported. It was observed that the elderly-onset PD group exhibited more DAT loss in the caudate nucleus and a higher risk of subsequent gait freezing than young-onset PD [74]. Furthermore, older adults with mild parkinsonism but not diagnosed as PD demonstrated decreased DAT binding in the caudate nucleus and anterior putamen. Additionally, the posterior putamen exhibited reduced DAT binding, falling between the levels observed in PD and healthy control subjects [75].

In summary, [^18^F]FP-CIT PET is a valuable biomarker for monitoring the dopaminergic system; furthermore, the decrease in DAT binding caused by neurodegeneration shows a more selective pattern in the posterior putamen, and aging seems to affect the whole striatum. Elderly patients with parkinsonism appear more likely to have additional motor symptoms as DAT binding in the caudate nucleus is also reduced in addition to the posterior putamen. These research results will be the basis for the normal range of [^18^F]FP-CIT PET by age group and the determination of normal or abnormal by quantitative analysis.

### Factors Affecting DAT Binding

Many researchers have attempted to understand various clinical-social and laboratory factors' effects on FP-CIT uptake. Bilirubin is one of the anti-oxidative molecules, and it is known that the bilirubin level is elevated in patients with PD compared to the control group and shows a positive correlation with the posterior putaminal uptake of the PD group [76]. Glucose loading correlated with DAT binding of caudate nucleus and putamen but no correlation with ventral striatum, showing subregional variability [77, 78]. There was a significant positive correlation between high-density lipoprotein cholesterol and DAT binding of the caudate nucleus [56]. Even a few clinical-social factors may affect striatal DAT binding. Current smokers showed higher DAT binding in the posterior and ventral putamen than ex-smokers and non-smokers, but no additional clinical benefits were observed [79]. It has been reported that a history of cancer is related to the motor reserve of patients with PD. UPDRS motor score was lower in the group with a history of cancer than the group with no premorbid cancer history; even [^18^F]FP-CIT uptake in the posterior putamen was similar; this suggests the possibility of a protective effect of the history of cancer on PD [80]. A recent longitudinal study also discussed the inverse relationship between PD and cancer in Korea [81]. Patients with PD with a higher education level (over 12 years) of similar symptom duration showed lower motor deficits than patients with a lower education level, even though DAT binding of the posterior putamen was lower on [^18^F]FP-CIT PET [82].

As described in the "Patient preparation and precautions" section, various drugs can affect the DAT binding of FP-CIT. These drugs can be broadly categorized into those that significantly affect visual analysis and those that, while not to a significant degree, exert an influence at the quantitative level. Reports on drugs affecting FP-CIT uptake have mainly focused on [^123^I]FP-CIT [83, 84]. Since drug interactions influencing DAT binding are primarily related to the characteristics of FP-CIT, it is expected that there won't be a significant difference between [^123^I]FP-CIT and [^18^F] FP-CIT. For patients taking anti-parkinson medication including levodopa, dopamine agonists, COMT inhibitors, and MAO-B inhibitors, it is generally known that it does not significantly impact FP-CIT uptake [11, 85–88], although minor effects may exist in quantitative analysis or research settings. Drugs relatively well-known to affect FP-CIT uptake include cocaine, amphetamines, phentermine, ephedrine, dexamphetamine, modafinil, and methylphenidate. These CNS stimulants used for ADHD, appetite suppression, narcolepsy exhibit high affinity for DAT, leading to a severe decrease of FP-CIT uptake [89–92]. The effects of methylphenidate have been reported not only in [^123^I]FP-CIT SPECT [93] but also in [^11^C] altropane PET [94] and [^99m^Tc]TRODAT-1 [95]. Modafinil is known to significantly influence visual analysis using PET agents such as [^11^C]PE2I [96] and [^11^C] altropane [97].

Other drugs, such as quetiapine, olanzapine, risperidone (antipsychotics), antidepressants (bupropion), opioids (fentanyl, codeine), have been reported to have no significant effects [98–100]. Anticholinergic drugs (benztropine, trihexyphenidyl), and cholinesterase inhibitors used for symptom control may have an impact on quantification, but there have been no reports with significant effects on visual analysis.

[^18^F]FP-CIT has an affinity for SERT and DAT. Still, there was no significant difference in striatal uptake ratio according to the administration of selective serotonin reuptake inhibitor (SSRI), even though it has been reported that [^123^I]FP-CIT uptake was slightly increased by SSRI administration [101]. The uptake ratios of raphe nuclei decreased in the group administered SSRIs, but there was no significant difference in occipital cortex and striatal uptake ratios. So, for the quantification analysis of patients taking SSRI, the occipital cortex is recommended as a reference region [101].

Most of these factors, with a few exceptions, do not seem to have a significant effect on visual interpretation, as they affect uptake across the whole striatum rather than subregional effects. However, it is proposed to consider these factors regarding the quantitative analysis and the reference values.

### Correlation with Clinical Symptoms

PD includes cardinal movement symptoms (gait disturbance, bradykinesia, rigidity, resting tremor) and non-motor symptoms (NMS), including cognitive dysfunctions. Various studies reporting associations between clinical symptoms in PD and [^18^F]FP-CIT PET have been performed. In particular, associations between striatal subregions and various clinical symptoms have been reported based on the improved resolution of PET compared to SPECT.

#### Motor Symptoms

Several quantitative parameters obtained from [^18^F]FP-CIT PET imaging demonstrate correlations with disease severity, Hoehn and Yah stage, and clinical symptoms in patients with PD [17, 42, 102, 103]. In the 3D analysis of [^18^F]FP-CIT PET, each striatal subregion showed correlations with each item of the UPDRS motor score. Particularly, axial and akinetic-rigidity PD symptoms showed the highest correlation with the uptake of the left caudate nucleus [104]. It has been reported that the sequence effect of the more affected hand, in which the speed and amplitude of repetitive actions gradually decrease in bradykinesia, has a negative correlation with DAT binding of the caudate nucleus evaluated by [^18^F]FP-CIT PET [105]. Putamen uptake on [^18^F]FP-CIT PET showed a correlation with mean speed and amplitude, and caudate nucleus uptake showed a correlation with the variability of amplitude and speed in a study that measured the amplitude, speed, and frequency of finger tapping in patients with PD using a gyroscope [106]. [^18^F]FP-CIT PET findings also appear differently depending on the pattern of gait disturbance. DAT binding of the posterior putamen is more reduced in the group with reduced cadence compared to the group with reserved cadence [107]. It is known that the striatal subregional pattern is also associated with the phenotype of MSA. The DAT loss pattern was reported to be associated with levodopa response, urinary incontinence, and age of onset by principal component analysis [108]. Pre-synaptic MSA with decreased DAT binding of posterior putamen predominantly showed milder parkinsonian features but more common and severe cerebellar ataxia than the trans-synaptic MSA type, which also showed a reduced metabolism in [^18^F]FDG PET [109].

#### Non-motor Symptoms

NMS can present in many different ways, such as fatigue, orthostatic hypotension, olfactory dysfunction, dysphagia, sleep disorders, memory impairment, depression, and hallucination, and substantially affect the quality of life of patients with PD. It was found that patients with overall DAT loss of associative/limbic striatum in [^18^F]FP-CIT PET had a higher NMS burden [110], and associations between subregional DAT binding patterns and several NMS have been reported. There was a controversy about autonomic dysfunction in PD. In a study comparing [^18^F]FP-CIT PET with orthostatic hypotension, a common autonomic dysfunction in patients with PD, there was no significant difference in striatal DAT binding depending on the presence or absence of orthostatic hypotension [111]. However, in another study, the PD group with autonomic dysfunction showed more reduced DAT binding in all striatal subregions than PD without autonomic dysfunction. In this study, the uptake of the parieto-occipital cortex was reduced, and the uptake of the pallidothalamic, pontocerebellar, inferior frontal, and primary motor cortex was increased in the early-phase images [112]. Associations between [^18^F]FP-CIT PET and clinical symptoms have also been reported in rapid eye movement sleep behavior disorder (RBD) patients, considered the prodromal state of PD. Patients with PD with RBD showed more reduced DAT binding in the ventral striatum and anterior caudate on [^18^F]FP-CIT PET and revealed decreased attention function compared to PD without RBD. However, further studies are needed because these findings were not statistically significant when applying multiple test corrections [113]. In addition, it was found that there was a difference in [^18^F]FP-CIT uptake depending on whether or not olfactory impairment was accompanied in RBD patients. RBD patients with olfactory impairment showed decreased DAT binding in the anterior and posterior putamen, caudate, and substantia nigra than RBD patients without olfactory impairment [114]. Hyposmic iRBD patients showed decreased DAT binding in [^18^F]FP-CIT PET at the baseline and more rapid cognitive decline at the follow-up [115]. Similar to the results in RBD patients, DAT binding of the caudate nucleus was found to be more reduced in patients with early PD with olfactory dysfunction [116]. A study investigating the association between dysphagia assessed by videofluoroscopic swallowing study and [^18^F]FP-CIT PET revealed that sub-items of dysphagia were associated with decreased DAT binding of the ventral striatum and caudate nucleus [117].

#### Cognitive Function

An early study showed no difference in [^18^F]FP-CIT PET pattern between PD with and without dementia [118]. However, several subsequent studies reported associations between cognitive function and DAT binding pattern. Despite the same disease duration, patients with PD with mild cognitive impairment showed decreased DAT binding in all striatal subregions compared to PD with normal cognitive function [119]. In addition, non-demented patients with PD with amyloid PET positivity showed amnestic-type MCI and decreased DAT binding of the left ventral striatum on [^18^F]FP-CIT PET [120]. Although there is a controversy in Lewy body dementia patients [121], reduced striatal [^18^F]FP-CIT uptake was associated with attention, language, and frontal/executive dysfunction independently of amyloid deposition measured by [^18^F]florbetaben PET [122].

### Dopamine-dependent Functional Network Connectivity

The dopaminergic system of the human brain includes the four major systems: mesolimbic pathway, mesocortical pathway, nigrostriatal pathway, and tuberoinfundibular pathway. [^18^F]FP-CIT PET is used to evaluate the nigrostriatal system, and many studies have been conducted regarding the cortico-striatal connection. It was reported that dopaminergic input of the caudate nucleus in normal subjects and patients with early PD was related to cognitive function in the comparison study with [^18^F]FP-CIT PET and perfusion imaging using [^15^O]H_2_O PET [123]. The PD cognition-related metabolic pattern is reported as the association of metabolism in the left caudate nucleus and the right cortical hemisphere evaluated by [^18^F]FDG PET and also related with decreased DAT binding of the caudate nucleus in [^18^F]FP-CIT PET [124]. The metabolism in the posterior cerebral cortex evaluated by [^18^F]FDG PET is associated with DAT binding of all striatal subregions (P < 0.05) except the posterior putamen and correlated with cognitive dysfunction, specifically frontal/executive function and visuospatial function [125]. Decreased uptake observed in the prefrontal cortex during early perfusion imaging demonstrated a correlation with diminished DAT binding in the caudate nucleus during delayed imaging; this was particularly notable in the group of patients exhibiting cognitive dysfunction [126]. Furthermore, the PD cognition-related and PD-related patterns, a metabolic pattern shown in [^18^F]FDG PET of patients with PD, were similar in early perfusion [^18^F]FP-CIT PET, indicating that it can be replaced [28]. An analysis for cortico-striatal functional connectivity using functional MRI and [^18^F]FP-CIT PET revealed that the pattern of dopamine-dependent functional connectivity varied according to the striatal subregion. In particular, the dopamine-dependent structure functionally connected to the posterior putamen, where DAT binding is first reduced in PD, was mainly found in the cerebellum [127]. Significant correlations between striatal binding ratio and gray matter density were found in the cerebellum, parahippocampal gyri, and frontal cortex in a cortico-striatal circuit study using simultaneous [^18^F]FP-CIT PET/MR instead of separated functional MRI and [^18^F]FP-CIT PET [128]. In a network study using diffusion tensor imaging and [^18^F]FP-CIT PET in patients with PD, DAT binding in the caudate nucleus was correlated with frontal /executive function and white matter structural alteration [129]. In another study using diffusion tensor imaging and [^18^F]FP-CIT PET, the PD group showed that nigrostriatal dopaminergic degeneration was accompanied by structural changes in the primary motor cortex and its output pathway [130]. The motor reserve predicted by the residual model using [^18^F]FP-CIT PET was also correlated with the risk of verbal memory function and dementia conversion [131].

Thus, various reports on the nigrostriatal pathway and functional connectivity have been made through comparative studies with [^18^F]FP-CIT PET and perfusion/metabolism or structural imaging methods. Among them, cognitive and frontal/executive functions were mostly reported to be associated with the DAT binding in the caudate nucleus. On the other hand, the posterior putamen, the first affected subregional striatum in PD, seems to be associated with the cerebellar cortex.

### Machine Learning Application Using [^18^F]FP-CIT PET/CT

As we embrace the era of artificial intelligence, various deep learning technologies have found applications in medical imaging. Studies utilizing deep learning with [^18^F]FP-CIT PET have also been reported. In addition to deep learning algorithms, various conventional machine learning techniques are frequently applied to [^18^F]FP-CIT PET. In the texture analysis studies, 3D-based metrics such as normalized contrast, code-similarity, and contrast could better distinguish PD from HC than two-dimensional-based metrics, and several machine learning models with those features including gradient boosting (95.7%), random forest (88.4%), and logistic regression (91.3%) showed high accuracy [132]. Convolutional neural network-based model using only MIP images instead of 3D orthogonal images performed similarly to nuclear medicine physicians in discriminating between PD and non-PD [133]. Not only for clinical diagnosis; artificial intelligence is also applied for imaging generation or reconstruction. Applying the attenuation correction method using a convolutional neural network model showed less noisy and more uniform u-maps in the [^18^F]FP-CIT PET images obtained from the time-of-flight PET system [134]. A study was conducted to investigate the heterogeneity of parkinsonism by applying principal component analysis and singular value decomposition, one of the machine learning algorithms, to [^18^F]FP-CIT PET imaging and performed unsupervised clustering [135].

Machine learning algorithms have recently been used in more diverse fields, such as automatic VOI generation, image quality improvement, early-to-delay image generation, and image classification. More research and attention are needed to improve patient convenience and the quality of imaging using these deep learning algorithms.

### Other Movement Disorders

A report stated that DAT binding of [^18^F]FP-CIT in bilateral putamen was reduced in amyotrophic lateral sclerosis patients with parkinsonism [136]. In motor neuron disease (MND) patients, the PD-like MND group with asymmetric resting tremor dominance and responsiveness to levodopa revealed decreased DAT binding, mainly in the dorsolateral putamen. In contrast, DAT binding of the caudate nucleus was also reduced in the parkinsonism-plus syndrome-like MND group with symmetric, akinetic rigidity and postural instability dominance and unresponsiveness to levodopa [137]. Perry syndrome, characterized by autosomal dominant parkinsonism, depression, and weight loss, is caused by a *DCTN1* gene mutation and shows severe DAT binding loss in [^18^F]FP-CIT PET, even when mild parkinsonism is accompanied [138]. McLeod syndrome is a rare X-linked recessive genetic disorder presenting as generalized chorea and dystonia. A case report found a hemizygous mutation of the *XK* gene (c.856_860delCTCTA; p.Leu286TyrfsTer16) through whole exome sequencing and DAT binding in the bilateral caudate nucleus and putamen was reduced in [^18^F]FP-CIT PET. Brain metabolism evaluated by [^18^F]FDG PET exhibited decreased metabolism, and MRI showed atrophic changes in the corresponding area [139]. Dual-phase [^18^F]FP-CIT PET performed in a Huntington’s disease patient with a confirmed CAG 18/43 repeats mutation showed reduced early imaging uptake in the basal ganglia and frontal-parietal-temporal lobe. It decreased DAT binding in ventral and posterior putamen in delayed imaging. Concomitant [^99m^Tc]ethyl cysteinate dimer (ECD) SPECT also showed findings similar to those of early perfusion imaging [140].

Several cases of Creutzfeldt-Jakob disease (CJD) have utilized [^18^F]FP-CIT PET for diagnosis. The dual-phase [^18^F]FP-CIT PET approach has proven more effective in the early diagnosis of CJD compared to diffusion-weighted imaging (DWI) MRI. In the Heidenhain variant CJD patient, delay imaging showed normal findings, but the left parieto-occipital cortex exhibited decreased uptake in the early phase, which was not observed on MRI at that time. Ultimately, a follow-up MRI showed high signal intensity in the affected area. In another patient with corticobasal syndrome, bilateral occipito-parietal cortex, basal ganglia, and thalamus revealed decreased uptake in the early phase and diffusely decreased striatal DAT binding in the delay phase. At that time, no abnormal findings were evident on DWI MRI, but high SI was observed in the cortex and basal ganglia on the follow-up DWI MRI imaging [141, 142].

### Future Applications

With the advent of next-generation sequencing, numerous genetic abnormalities associated with parkinsonism have been identified. [^18^F]FP-CIT PET can reveal specific patterns in patients with different genetic mutations, aiding in diagnosing and understanding disease progression [143–150]. In the whole exome sequencing study conducted on the Korean (*PLA2G6*-associated neurodegeneration) PLAN family, proband with a heterozygous mutation of the *PARK15* gene presented adult-onset dystonia-parkinsonism as clinical symptom and [^18^F]FP-CIT PET showed a severe decrease in the whole putamen. But the affected brother in whom non-synonymous single nucleotide variants in a gene related to iron accumulation were found, presented childhood-onset atypical neuroaxonal dystrophy and [^18^F]FP-CIT PET finding was normal [143]. Parkin type of early-onset Parkinson disease (PARK-*Parkin*) with parkin mutation showed a slightly more symmetric decrease in DAT binding of putamen compared to patients with non-parkin mutation EOPD, and a significant decrease in anteroventral putamen compared to posterodorsal portion [151].

[^18^F]FP-CIT PET can be a valuable tool for assessing treatment response in PD. Studies have shown changes in tracer uptake following various treatments, including cell transplantation and drug therapies, offering a non-invasive way to evaluate therapeutic efficacy [152–159]. [^18^F]FP-CIT uptake of injured striatum was increased after co-transplantation of schwann cells (SCs) and human embryonic nerve stem cells (NSCs) in Macaque PD [152]. It was reported that [^18^F]FP-CIT PET can be used to exhibit treatment effect along with behavioral improvement by NSC transplantation in a 6-OHDA-injected mouse model [153]. Recombinant human erythropoietin therapy showed improvement in non-motor symptoms (NMS) of PD patients and [^18^F]FP-CIT PET was used to evaluate treatment response in this study [154]. After bone marrow mesenchymal stem cell (BMSC) transplantation in a rat model, improvement of symptoms and recovery of DAT binding on [^18^F]FP-CIT PET were confirmed [155].As it is a neurodegenerative disease with the second highest prevalence after Alzheimer’s, numerous studies are being conducted on treating PD, and [^18^F]FP-CIT PET is expected to serve as a valuable biomarker for evaluating the response of these therapeutic strategies.

Finally, numerous studies have reported the utility of DAT imaging in detecting preclinical PD in patients with hyposmia or RBD [160–164]. However, research using [^18^F]FP-CIT PET in such patients has been limited [113–116]. Further studies are needed to evaluate whether [^18^F]FP-CIT PET is useful in the early diagnosis of PD and in assessing high-risk groups in these patient populations.

## Conclusion

The review article provides an imaging procedure and systematic review of literatures for clinical applications of [^18^F]FP-CIT, a radiopharmaceutical used for DAT imaging via PET. [^18^F]FP-CIT PET is a valuable tool for detecting dopaminergic neuronal degeneration, aiding in the diagnosis of parkinsonian syndromes. [^18^F]FP-CIT PET holds promise for improving our understanding of parkinsonism and its applications in early diagnosis, treatment monitoring, and investigating the broader dopaminergic system. Further research in these areas will enhance its utility in clinical practice.

## Data Availability

Not applicable.
