# Correction to: Imaging Procedure and Clinical Studies of [^18^F]FP‑CIT PET

**DOI:** 10.1007/s13139-024-00881-2

**Published:** 2024-08-27

**Authors:** Changhwan Sung, Seung Jun Oh, Jae Seung Kim

**Affiliations:** Department of Nuclear Medicine, Asan Medical Center, University of Ulsan College of Medicine, 88, Olympic‑Ro 43‑Gil, 05505 Songpa‑Gu, Seoul, Republic of Korea

**Correction to Nuclear Medicine and Molecular Imaging (2024) 58:185–202**

10.1007/s13139-024-00840-x

The article Imaging Procedure and Clinical Studies of [^18^F]FP‑CIT PET, written by Changhwan Sung, Seung Jun Oh and Jae Seung Kim, was originally published Online First without Open Access. After publication in volume 58, issue 4, pages 185–202 the author decided to opt for Open Choice and to make the article an Open Access publication. Therefore, the copyright of the article has been changed to © The Authors, 2024 and the article is forthwith distributed under the terms of the Creative Commons Attribution (CC BY) license 4.0 International License, which permits use, sharing, adaptation, distribution and reproduction in any medium or format, as long as you give appropriate credit to the original author(s) and the source, provide a link to the Creative Commons licence, and indicate if changes were made. The images or other third party material in this article are included in the article’s Creative Commons licence, unless indicated otherwise in a credit line to the material. If material is not included in the article’s Creative Commons licence and your intended use is not permitted by statutory regulation or exceeds the permitted use, you will need to obtain permission directly from the copyright holder. To view a copy of this licence, visit http://creativecommons.org/licenses/by/4.0.

The original article has been corrected.

